# Supplementary material for: Diversity of the Bacterial Community Associated with Hindgut, Malpighian Tubules, and Foam of Nymphs of Two Spittlebug Species (Hemiptera: Aphrophoridae)
Source: Microorganisms. 2023 Feb 13;11(2):466. doi: 10.3390/microorganisms11020466 (PMC9967529; doi:10.3390/microorganisms11020466)
Supplement: Supplementary file 1 [file microorganisms-11-00466-s001.zip › Table S1.pdf]

Table S1. Results of BLAST search on sequenced 16S rDNA bands selected from PCR-DGGEs, the accession number of the nearest known bacterial species with a sequence coverage of 100% was reported. Taxonomic identification was achieved by using different sequence similarity thresholds: a similarity ≥97%, ≥95%, ≥90%, ≥85%, ≥80% and ≥75% for assignment at the species-, genus-, family-, order-, class- and phylum-levels identification, respectively [30]. YP, posterior tubular midgut; MG , filter chamber linked to the conical segment; MT, Malpighian tubules; IL, ileum.

Band	Sample	Nearest match (GenBank accession no.; % similarity)	Taxonomical identification
Ps-1	MG of *Philaenus spumarius*	*Sodalis praecaptivus* (AM237373; 97.4%)	*Sodalis praecaptivus*
Ps-2	MG of *Philaenus spumarius*	*Sodalis glossinidius* (LN854557; 97.9%)	*Sodalis glossinidius*
Ps-3	MG of *Philaenus spumarius*	*Sodalis glossinidius* (LN854557; 98.1%)	*Sodalis glossinidius*
Ps-4	MG of *Philaenus spumarius*	*Escherichia coli* (MN083301; 100%)	*Salmonella enterica*
Ps-5	MT of *Philaenus spumarius*	*Sodalis glossinidius* (LN854557; 97.9%)	*Sodalis glossinidius*
Ps-6	MT of *Philaenus spumarius*	*Rhodococcus gingshengii* (MN826591; 100%)	*Rhodococcus gingshengii*
Ps-9	MG of *Philaenus spumarius*	*Rhodococcus gingshengii* (MN826591; 100%)	*Rhodococcus gingshengii*
Ps-11	MT of *Philaenus spumarius*	*Escherichia coli* (MN083301; 100%)	*Salmonella enterica*
Lc-18	YP of *Lepyronia coleoptrata*	*Rickettsia bellii* (KU586119; 99.0%)	*Rickettsia bellii*
Lc-19	YP of *Lepyronia coleoptrata*	*Rickettsia bellii* (KU586119; 99.2%)	*Rickettsia bellii*
Lc-20	MT of *Lepyronia coleoptrata*	*Rickettsia bellii* (KU586119; 98.2%)	*Rickettsia bellii*
Lc-21	IL of *Lepyronia coleoptrata*	*Escherichia coli* (MN083301; 100%)	*Salmonella enterica*
Lc-22	IL of *Lepyronia coleoptrata*	*Rhodococcus gingshengii* (MN826591; 100%)	*Rhodococcus gingshengii*
Lc-23	MT of *Philaenus spumarius*	*Rhodococcus gingshengii* (MN826591; 100%)	*Rhodococcus gingshengii*
F-7	Foam of *Lepyronia coleoptrata*	*Sinorhizobium sp.* (CP044012; 96.5%)	*Sinorhizobium sp.*
F-9	Foam of *Lepyronia coleoptrata*	*Erwinia rhapontici* (MN826571; 99.7%)	*Erwinia rhapontici*
F-10	Foam of *Philaenus spumarius*	*Pigmentiphaga humi* (MH667611; 99.5%)	*Pigmentiphaga humi*
F-11	Foam of *Philaenus spumarius*	*Ciceribacter selenitireducens* (MH665748; 99.5%)	*Ciceribacter selenitireducens*
F-12	Foam of *Philaenus spumarius*	*Devosia oryziradicis* (CP068047; 97,2%)	*Devosia oryziradicis*
F-13	Foam of *Philaenus spumarius*	*Ciceribacter selenitireducens* (MH665748; 99.5%)	*Ciceribacter selenitireducens*
F-14	Foam of *Philaenus spumarius*	*Erwinia rhapontici* (MN826571; 99.7%)	*Erwinia rhapontici*
F-15	Foam of *Philaenus spumarius*	*Brevundimonas mediterranea* (MK250497; 99.7%)	*Brevundimonas mediterranea*
F-16	Foam of *Philaenus spumarius*	*Ciceribacter azotifigens* (KX510117; 97.6%)	*Ciceribacter azotifigens*
F-17	Foam of *Philaenus spumarius*	*Ciceribacter azotifigens* (KX510117; 97.6%)	*Ciceribacter azotifigens*
F-18	Foam of *Philaenus spumarius*	*Stenotrophomonas rhizoplilia* (MT078676; 100%)	*Stenotrophomonas rhizoplilia*
F-26	Foam of *Lepyronia coleoptrata*	*Brevundimonas mediterranea* (MK250497; 99.7%)	*Brevundimonas mediterranea*
F-28	Foam of *Lepyronia coleoptrata*	*Rhizobium skierniewicense* (MN826327; 99.7%)	*Rhizobium skierniewicense*
F-29	Foam of *Lepyronia coleoptrata*	*Rhizobium skierniewicense* (MN826327; 99.7%)	*Rhizobium skierniewicense*
F-31	Foam of *Philaenus spumarius*	*Ciceribacter azotifigens* (KX510117; 97.6%)	*Ciceribacter azotifigens*
