# Supplementary figures and images for: Diversity of the Bacterial Community Associated with Hindgut, Malpighian Tubules, and Foam of Nymphs of Two Spittlebug Species (Hemiptera: Aphrophoridae)
Source: Microorganisms. 2023 Feb 13;11(2):466. doi: 10.3390/microorganisms11020466 (PMC9967529; doi:10.3390/microorganisms11020466)

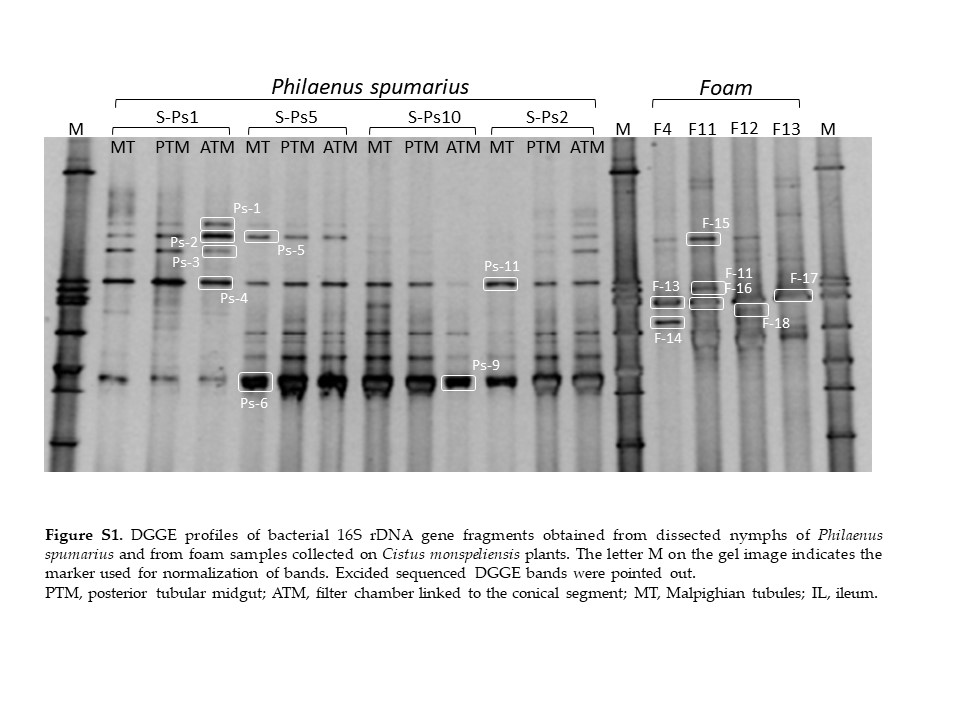

Supplement: Supplementary file 1 [file microorganisms-11-00466-s001.zip › Figure S1.jpg]

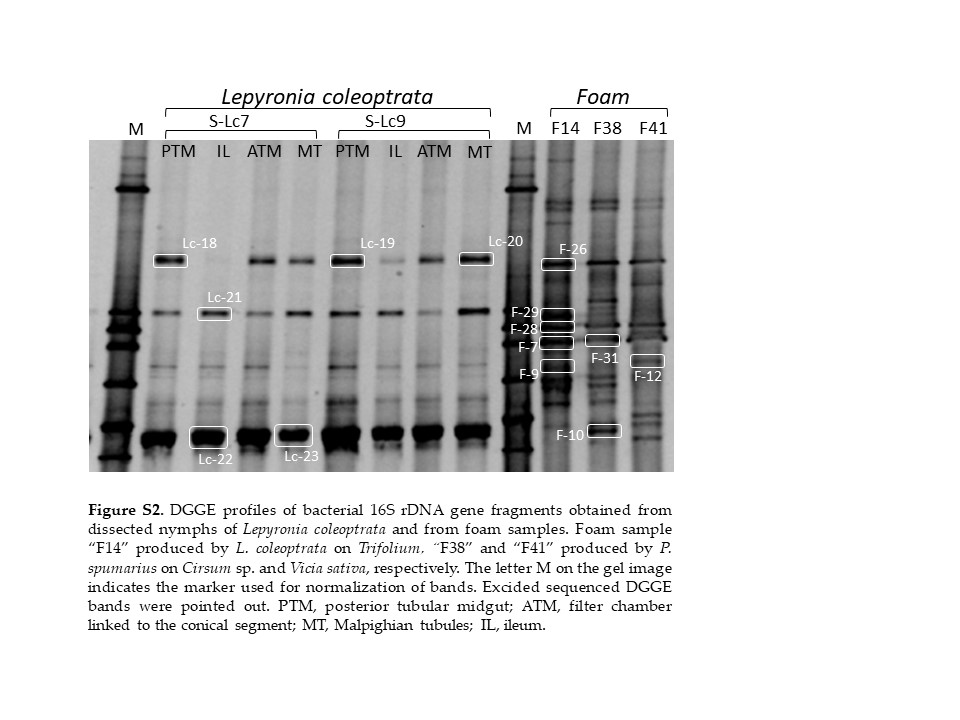

Supplement: Supplementary file 1 [file microorganisms-11-00466-s001.zip › Figure S2.jpg]

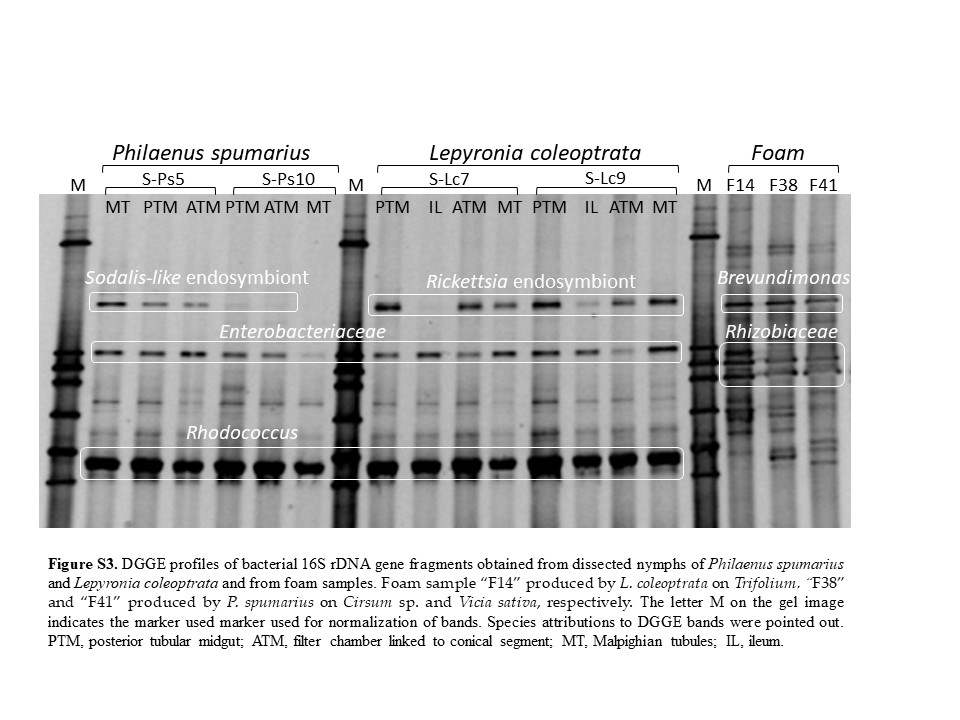

Supplement: Supplementary file 1 [file microorganisms-11-00466-s001.zip › Figure S3.jpg]

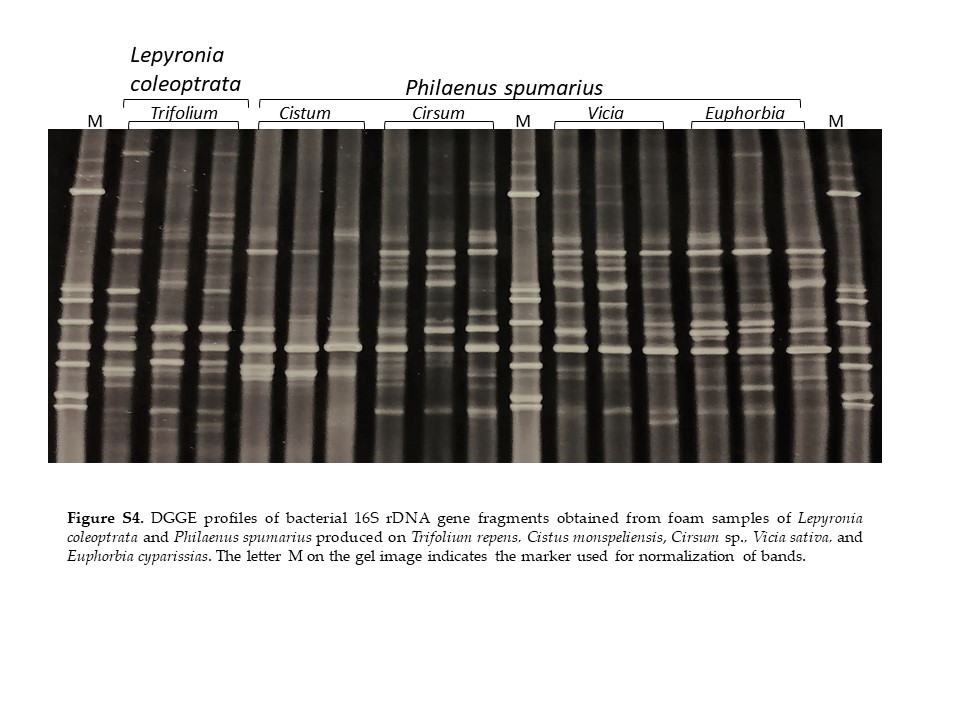

Supplement: Supplementary file 1 [file microorganisms-11-00466-s001.zip › Figure S4.jpg]
